# 803 Evaluation of Burn Depth and Reactive Inflammation Using Perioperative Fluorescence Imaging

**DOI:** 10.1093/jbcr/irae036.343

**Published:** 2024-04-17

**Authors:** Mary Junak, Jocelyn C Zajac, Aiping Liu, Sydney Jupitz, Trevor Seets, Tisha Kawahara,, Adam Uselmann, Christie Lin, Lauren B Nosanov, Lee D Faucher, Angela Gibson

**Affiliations:** University of Wisconsin, Fitchburg, WI; University of Wisconsin School of Medicine and Public Health, Madison, WI; University of Wisconsin-Madison, Madison, WI; OnLume, Madison, WI; OnLume Surgical, Madison, WI; OnLume Inc, Madison, WI; University of Wisconsin, Middleton, WI; University of Wisconsin, Madison, WI; University of Wisconsin, Fitchburg, WI; University of Wisconsin School of Medicine and Public Health, Madison, WI; University of Wisconsin-Madison, Madison, WI; OnLume, Madison, WI; OnLume Surgical, Madison, WI; OnLume Inc, Madison, WI; University of Wisconsin, Middleton, WI; University of Wisconsin, Madison, WI; University of Wisconsin, Fitchburg, WI; University of Wisconsin School of Medicine and Public Health, Madison, WI; University of Wisconsin-Madison, Madison, WI; OnLume, Madison, WI; OnLume Surgical, Madison, WI; OnLume Inc, Madison, WI; University of Wisconsin, Middleton, WI; University of Wisconsin, Madison, WI; University of Wisconsin, Fitchburg, WI; University of Wisconsin School of Medicine and Public Health, Madison, WI; University of Wisconsin-Madison, Madison, WI; OnLume, Madison, WI; OnLume Surgical, Madison, WI; OnLume Inc, Madison, WI; University of Wisconsin, Middleton, WI; University of Wisconsin, Madison, WI; University of Wisconsin, Fitchburg, WI; University of Wisconsin School of Medicine and Public Health, Madison, WI; University of Wisconsin-Madison, Madison, WI; OnLume, Madison, WI; OnLume Surgical, Madison, WI; OnLume Inc, Madison, WI; University of Wisconsin, Middleton, WI; University of Wisconsin, Madison, WI; University of Wisconsin, Fitchburg, WI; University of Wisconsin School of Medicine and Public Health, Madison, WI; University of Wisconsin-Madison, Madison, WI; OnLume, Madison, WI; OnLume Surgical, Madison, WI; OnLume Inc, Madison, WI; University of Wisconsin, Middleton, WI; University of Wisconsin, Madison, WI; University of Wisconsin, Fitchburg, WI; University of Wisconsin School of Medicine and Public Health, Madison, WI; University of Wisconsin-Madison, Madison, WI; OnLume, Madison, WI; OnLume Surgical, Madison, WI; OnLume Inc, Madison, WI; University of Wisconsin, Middleton, WI; University of Wisconsin, Madison, WI; University of Wisconsin, Fitchburg, WI; University of Wisconsin School of Medicine and Public Health, Madison, WI; University of Wisconsin-Madison, Madison, WI; OnLume, Madison, WI; OnLume Surgical, Madison, WI; OnLume Inc, Madison, WI; University of Wisconsin, Middleton, WI; University of Wisconsin, Madison, WI; University of Wisconsin, Fitchburg, WI; University of Wisconsin School of Medicine and Public Health, Madison, WI; University of Wisconsin-Madison, Madison, WI; OnLume, Madison, WI; OnLume Surgical, Madison, WI; OnLume Inc, Madison, WI; University of Wisconsin, Middleton, WI; University of Wisconsin, Madison, WI; University of Wisconsin, Fitchburg, WI; University of Wisconsin School of Medicine and Public Health, Madison, WI; University of Wisconsin-Madison, Madison, WI; OnLume, Madison, WI; OnLume Surgical, Madison, WI; OnLume Inc, Madison, WI; University of Wisconsin, Middleton, WI; University of Wisconsin, Madison, WI; University of Wisconsin, Fitchburg, WI; University of Wisconsin School of Medicine and Public Health, Madison, WI; University of Wisconsin-Madison, Madison, WI; OnLume, Madison, WI; OnLume Surgical, Madison, WI; OnLume Inc, Madison, WI; University of Wisconsin, Middleton, WI; University of Wisconsin, Madison, WI

## Abstract

**Introduction:**

Early determination of burn depth is essential for guiding proper treatment of burn injuries. The primary technique used to evaluate burn depth is visual assessment, relying heavily on subjective interpretation while risking over-excision. Second window indocyanine green (SWIG) is a novel method of delayed fluorescence imaging with possible utility in burn surgery, as indocyanine green (ICG) persists in burn wounds 24 hours after intravenous injection. The objective of this study is to correlate intraoperative SWIG florescence with burn depth as evaluated by lactate dehydrogenase (LDH) staining. Additionally, given inflammation is thought to impact the progression of a burn, we investigated the relationship between SWIG florescence signal and reactive inflammation.

**Methods:**

Consented patients with indeterminate depth burns received a 5 mg/kg ICG infusion during their third daily burn care after admission or 24-hours prior to surgery. SWIG was performed 24 hours after ICG injection on a region of interest (ROI) during wound care or during burn excision. A full thickness skin biopsy was taken from the center of the ROI and processed for ICG microscopy and staining. Burn depths were scored using LDH-stained sections. To investigate the relationship between SWIG and inflammation, nude mice underwent a full thickness contact burn or received endotoxin to induce inflammation. Mice were injected with ICG at 5mg/kg and SWIG signal was captured at the non-burned control, burned, and inflamed ROIs 24 hours after injection. Signal-background ratio (SBR) was calculated using the ratio between the averaged ICG intensity in the burned or inflamed ROI and the control ROI. GraphPad Prism 8.0 was used for statistical analyses.

**Results:**

There was no correlation between the raw SWIG fluorescence value of the ROI in vivo and burn depth as determined by LDH staining. Furthermore, when the fluorescence value of the ROI was normalized to the SWIG intensity of the overall image and the SWIG intensity of non-burn control skin, there was no correlation to burn depth. In mice, SWIG SBR was significantly higher in the burned region (3.2±0.5) compared to the endotoxin induced inflamed regions (1.6±0.1).

**Conclusions:**

There is heterogeneity in the intraoperative fluorescence signal when compared to burn depth. This variability in fluorescence signal could be attributed to the effects of local inflammation on the burn wound microenvironment as animal studies have shown that ICG signal is present in non-burn inflamed tissue. Further studies are warranted to discern the role of inflammation on burn wound progression and fluorescence signal in humans.

**Applicability of Research to Practice:**

This data supports the potential utility of perioperative ICG fluorescence imaging for guidance of surgical excision in patients with indeterminate depth burns.

